# Investigation on the Thermal Behavior, Mechanical Properties and Reaction Characteristics of Al-PTFE Composites Enhanced by Ni Particle

**DOI:** 10.3390/ma11091741

**Published:** 2018-09-16

**Authors:** Jiaxiang Wu, Huaixi Wang, Xiang Fang, Yuchun Li, Yiming Mao, Li Yang, Qin Yin, Shuangzhang Wu, Miao Yao, Jiaxing Song

**Affiliations:** 1 College of Field Engineering, PLA Army Engineering University, Nanjing 210001, China; wujiaxiang1356@163.com (J.W.); fangxiang3579@163.com (X.F.); liyuchunmail@163.com (Y.M.); speedinessli@163.com (L.Y.); dfyq1982@21.com (Q.Y.); wushzh4394@126.com (S.W.); yaoshunmiao@126.com (M.Y.); 18351950537@163.com (J.S.); 2 31104 Troop of PLA, Nanjing 210001, China; whx827765479@163.com

**Keywords:** Al-Ni-PTFE, thermal behavior, mechanical properties, reaction characteristics, DSC, quasi-static compression

## Abstract

Al-PTFE (aluminum-polytetrafluoroethene) is regarded as one of the most promising reactive materials (RMs). In this work, Ni (Nickel) was added to Al-PTFE composites for the purpose of improving the energy density and damage effect. To investigate the thermal behavior, mechanical properties and reaction characteristics of the Al-Ni-PTFE composites, an Al-PTFE mixture and an Al-Ni mixture were prepared by ultrasonic mixing. Six types of Al-Ni-PTFE specimens with different component mass ratios were prepared by molding sintering. Simultaneous thermal analysis experiments were carried out to characterize the thermal behavior of the Al-PTFE mixture and the Al-Ni mixture. Quasi-static compression tests were performed to analyze the mechanical properties and reaction characteristics of the Al-Ni-PTFE specimens. The results indicate that the reaction onset temperature of Al-Ni (582.7 °C) was similar to that of Al-PTFE (587.6 °C) and that the reaction heat of Al-Ni (991.9 J/g) was 12.5 times higher than that of Al-PTFE (79.6 J/g). With the increase of Ni content, the material changed from ductile to brittle and the strain hardening modulus and compressive strength rose first and then subsequently decreased, reaching a maximum of 51.35 MPa and 111.41 MPa respectively when the volume fraction of Ni was 10%. An exothermic reaction occurred for the specimens with a Ni volume fraction no more than 10% under quasi-static compression, accompanied by the formation of Ni-Al intermetallic compounds. In the Al-Ni-PTFE system, the reaction between Al and PTFE preceded the reaction between Al and Ni and the feasibility of increasing the energy density and damage effect of the Al-Ni-PTFE reactive material by means of Ni-Al reaction was proved.

## 1. Introduction

Reactive materials, which are generally composed of two or more non-explosive solid materials, are a new type of energetic material induced by an impact. They stay inert under a normal temperature and pressure and undergo a violent reaction under impact while generating new substances and releasing a large amount of heat [1,2,3]. As a typical reaction material, Al-PTFE (aluminum-polytetrafluoroethene) has the characteristics of a higher energy density, better mechanical properties, stronger stability, and easier preparation compared with traditional energetic materials such as explosives and propellants [4,5]. In this regard, it has a great application value and development prospects in the military. It can be made into a high-energy warhead with an impact-reaction secondary damage in the form of an energetic liner or reaction fragment while penetrating the target by its own kinetic energy. Chemical reactions such as explosions and combustion will be triggered so as to achieve damage to the target [6,7].

Whether the Al-PTFE reactive material can obtain a good application depends on if it has sufficient strength to ensure its safety under the conditions of production, processing, storage and explosive loading and a chemical reaction can occur when penetrating the target. Therefore, in order to improve the strength and density of the materials, experimental research has been conducted worldwide to primarily focus on the engineering strain of Al-PTFE under different influence factors and the performance change after adding different fillers. Feng found that an Al-PTFE specimen, through a special heat treatment, could react violently under quasi-static compression for the first time and on this basis, the effects of the sintering temperature, the material ratio, and Al particle size on the quasi-static reaction of Al-PTFE were ascertained [8,9]. Wu studied the impact exerted by Al particle size on the mechanical properties and reaction characteristics of Al-PTFE. It was found that with the increase in Al particle size, the strength and sensitivity of the material decreased and the activation reaction of Al and PTFE became more and more difficult to occur. The formation of circumferential open cracks was a prerequisite for the Al-PTFE specimens to undergo a reactive reaction [10]. Cai investigated the mechanical properties of Al-W-PTFE materials and found that the addition of W particles could effectively improve the strength of Al-PTFE [11]. Herbold studied the effect of W particle size on the strength, failure and shock behavior of Al-W-PTFE composites by means of experimentation and simulation. The results indicated that the strength of Al-W-PTFE increased with a decrease in the W particle size [12]. Yu conducted quasi-static compression experiments and discovered that the addition of TiH2 particles to Al-PTFE can significantly enhance the strength of the composite and a special flame, different from the Al-PTFE reaction, was observed in the reaction phenomenon [13].

The previous research mainly focused on the performance change of Al-PTFE added to W particles and there is little research on the performance change after adding Ni particles [14,15,16]. Although W has an extremely high density (19.3 g/cm^3^), since W does not participate in the reaction, it is only used as a carrier for mass addition to Al-PTFE the energy density of the material is inevitably lowered under the condition that the volume fraction of the material is constant. Ni has a higher density (8.9 g/cm^3^) and Eakins found that Ni and Al can undergo chemical reactions under impact loading to form Ni-Al intermetallic compounds [17,18,19]. In this regard, the addition of Ni particles to Al-PTFE not only can increase the strength and density of the material but can also increase the energy density of the material and enhance the damaging effect on target. In this paper, the thermal behavior of an Al-PTFE mixture and an Al-Ni mixture were characterized by thermogravimetric-differential scanning calorimetry (TG-DSC) and six types of Al-Ni-PTFE specimens with different equivalence ratios were prepared. Their mechanical properties and reaction characteristics under quasi-static compression were investigated.

## 2. Experimental Section

### 2.1. Materials

The initial powders adopted to carry out the experiments were of the following average size: PTFE: 25 μm (from 3M, Shanghai, China); Al: 1–2 μm (from JT, Hunan, China); Ni: 2 μm (from Naiou, Shanghai, China).

### 2.2. Specimen Preparation

For thermal analysis, the mass ratios between Al and PTFE were set to 26.5%/73.6% (the chemical equilibrium ratio) and Al and Ni were mixed according to mass ratio 31%:69% (mole ratio 1:1) using the ultrasonic mixing method. Two kinds of mixtures were sonicated for 30 min in a sonic bath to prepare a homogeneously dispersed solution and then dried in a vacuum oven for 24 h at 60 °C.

**Figure 1:**
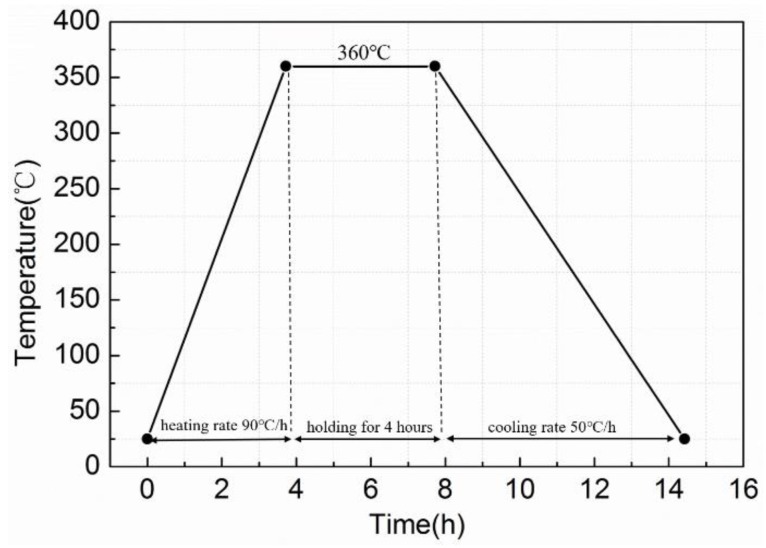
The temperature history of the sintering cycle.

For the quasi-static compression, six types of Al-Ni-PTFE specimens with different equivalence ratios were prepared. Table 1 lists the equivalence ratio and the theoretical maximum density (TMD) for the six types of Al-Ni-PTFE specimens; a type A specimen (Al/Ni/PTFE: 22 vol %/0/78 vol %) was prepared to serve as a reference. The preparation process of the specimens included mixing, cold isostatic pressing and vacuum sintering, which was based on the patent of Nielson [20]. The procedure was as follows: The Al-PTFE mixture was stirred using a motor-driven blender for 20 min in an ethanol solution, then put into a vacuum drying oven for 48 h at 60 °C and cold pressed into cylinders with sizes of Φ10 mm × 10 mm under a compressive pressure of 300 MPa. Finally, the pressed specimens were heated in a vacuum oven at the sintering temperature of 360 °C for 4 h with a heating rate of 90 °C·h^−1^ and a cooling rate of 50 °C·h^−1^. The temperature history of the sintering cycle is depicted in Figure 1.

### 2.3. Experimental Procedures

An investigation into the thermal behavior was carried out using a TG/DSC simultaneous thermal analyzer. The average sample mass was about 2.0 mg the and heating rate was 5 °C/min, covering the temperatures from 20 to 1000 °C. Argon was used as the insured gas with a flow rate of 30 mL/min.

**Figure 2:**
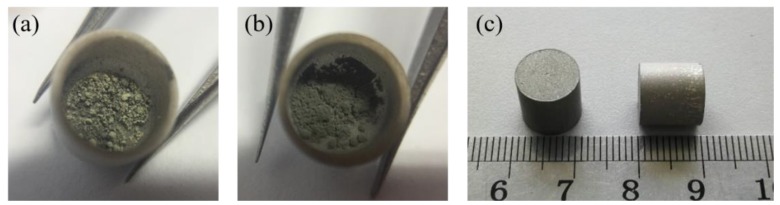
The samples used for DSC (differential scanning calorimetry) and quasi-static compression: (**a**) The Al-PTFE (aluminum-polytetrafluoroethene) mixture used for DSC; (**b**) the Al-Ni mixture used for DSC; (**c**) the Al-Ni-PTFE specimen used for quasi-static compression.

Quasi-static compression tests were performed using a universal materials testing machine (CMT5105, MTS, Eden Prairie, MN, USA) with a maximum loading capacity of 100 kN. A load was applied at a speed of 6 mm·min^−1^ corresponding to a nominal strain rate of 0.01 s^−1^. Prior to the tests, both ends of the specimen were lubricated with petroleum jelly aiming to alleviate the impact of friction. Each type of specimens was tested three times for the sake of guaranteeing the reliability of the experimental results. To observe the reaction process of six types of specimens under quasi-static compression more accurately, time sequences of the reaction process were captured using a high-speed camera (FASTCAM SA-Z, Photron, Tokyo, Japan) with the frame rate of up to 10,000 frames/s. In addition, the residues of the specimens after quasi-static compression were characterized by X-ray diffraction (Bruker D8 ADVANCE, Bruker, Berlin, Germany). A S-3400N II scanning electron microscope (SEM) (Hitachi, Tokyo, Japan) was used to observe the interior microstructures of the specimens before quasi-static compression. Figure 2 shows the samples used for DSC and quasi-static compression.

## 3. Results and Discussion

### 3.1. Mesoscale Characteristics

**Figure 3:**
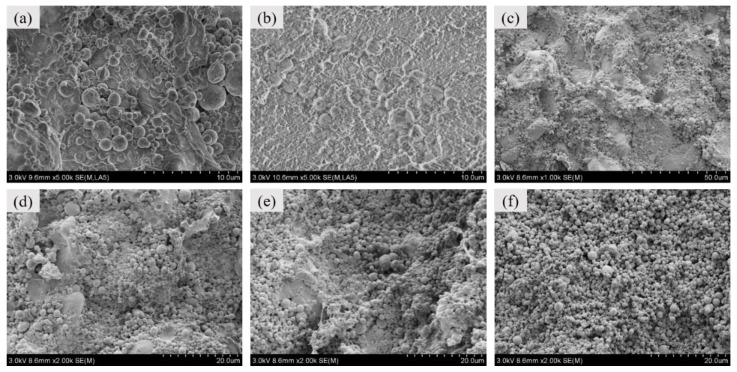
The interior microstructures of the specimens: (**a**) The type A specimen before sintering; (**b**) the type A specimen after sintering (local); (**c**) the type A specimen after sintering (overall); (**d**) the type B specimen after sintering; (**e**) the type C specimen after sintering; (**f**) the type F specimen after sintering.

The interior microstructures of the specimens are shown in Figure 3. Figure 3a,b compare the local interior microstructural characteristics of the type A specimen before sintering and after sintering in the case of the same magnification. Plainly, there was a crowd of voids and pores existing between the Al particles and the PTFE matrix before sintering. On the contrary, it was distinctly observed that the Al particles were embedded in the PTFE matrix very tightly without voids after sintering, as shown in Figure 3b. In this regard, the main function of the sintering was to obtain a cross-linking of the polymeric material and to permit the particles to fuse together to form a homogeneous material. After sintering, the mechanical properties of the specimen were significantly improved by virtue of the recrystallization of PTFE.

Figure 3c–f illustrates the overall interior microstructural characteristics of the type A, B, C and F specimens after sintering. Al and Ni can be discriminated facilely from the PTFE matrix due to their spherical geometry. It was observed that the Al, Ni and PTFE powders have been homogeneously mixed through the preparation process used in this paper. With the increase in the Ni content, the color of the SEM images became deeper owing to the Ni particles as brighter features compared to the Al particles arising from the lower atomic number. As can be seen from Figure 3c–e, when the content of Ni was relatively low, Al and Ni were well embedded in the PTFE matrix and played the role of supporting the matrix. However, excessive Ni destroyed the continuity of the PTFE matrix, which can be observed in Figure 3f.

### 3.2. Thermal Behavior of Al-PTFE and Al-Ni Mixtures

**Figure 4:**
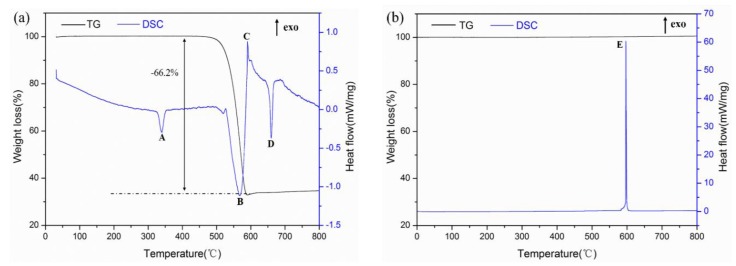
The TG-DSC curves for the Al-PTFE and Al-Ni mixtures: (**a**) The Al-PTFE mixture; (**b**) the Al-Ni mixture.

The DSC curves for the Al-PTFE and Al-Ni mixtures are presented in Figure 4 and all thermal analyses are summarized in Table 2. As can be seen from Figure 4a, there were three endothermic peaks and one exothermic peak in Figure 4a. The endo-peak-A started at 328.9 °C and the TG curve showed no mass change. It can be obtained that the endo-peak-A was the melting endothermic peak of PTFE. The endo-peak-B started at 502.2 °C, accompanied by a decrease in the sample quality, indicating the formation of gases, which can be judged as the decomposition of PTFE. The exo-peak-C began at 587.6 °C and was judged to be an exothermic reaction between the Al and the decomposition product of PTFE; the reaction heat was 79.6 J/g. The endo-peak-D started at about 649.1 °C, which was the melting endothermic peak of the incompletely reacted Al powder and the sample quality did not change during this period. According to Figure 4b, it can be seen that there was only one exothermic peak in during the whole heating process, implying that there was a reaction between Al and Ni starting at 582.7 °C and that there was no residual Al powder, otherwise there would have been an endothermic peak for the Al powder melting at about 660 °C. In the case of the same heating rate, the reaction heat of Al-Ni was 991.9 J/g, which was 12.5 times higher than that of Al-PTFE.

### 3.3. Mechanical Properties under Quasi-Static Compression

**Figure 5:**
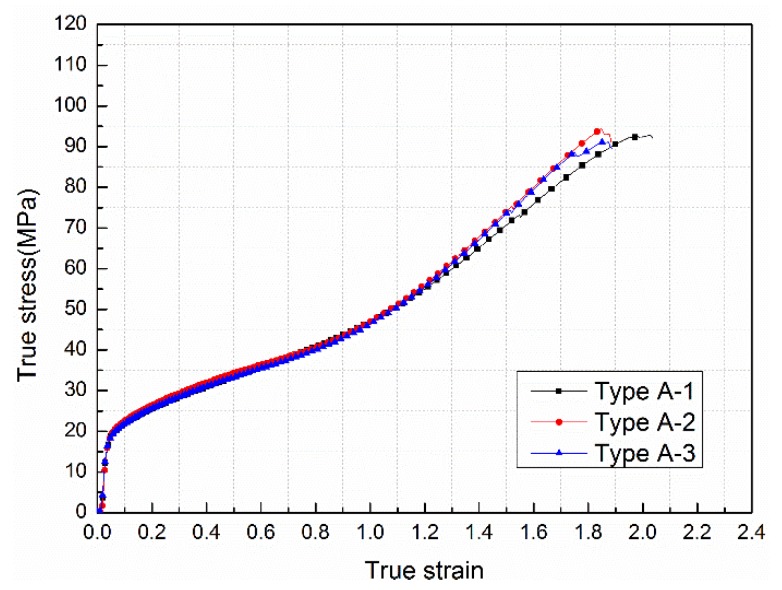
The true stress-strain curves for the type A specimen from triplicate experiments.

Take the experimental results of the type A specimen, for example, the true stress-strain curves obtained by triplicate experiments almost overlapped together and took on a high degree of agreement as shown in Figure 5, which implied that the testing data were of good consistency.

**Figure 6:**
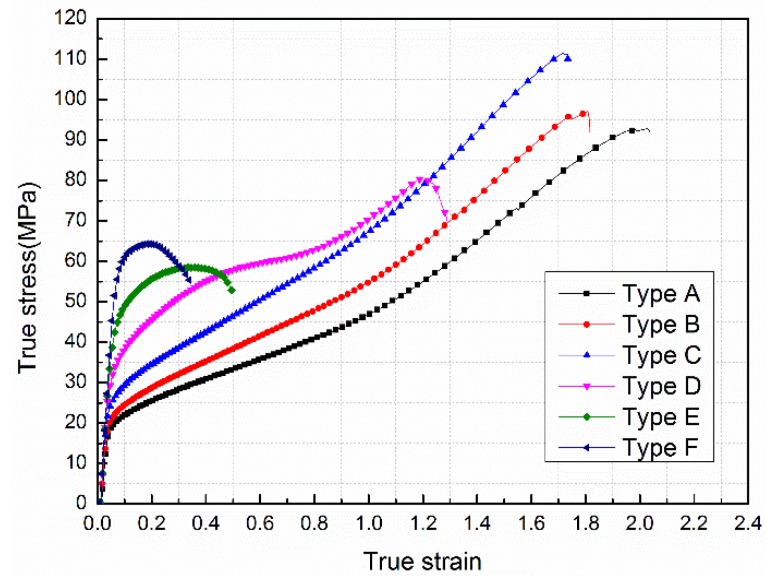
The true stress-strain curves for the six types of Al-Ni-PTFE specimens under quasi-static compression.

Figure 6 sketches the true stress-strain curves for the six types of Al-Ni-PTFE specimens under quasi-static compression. As shown in Figure 6, the six types of Al-Ni-PTFE specimens all experienced a linear elastic stage under quasi-static compression and the elastic moduli were similar. This phenomenon was owing to the fact that the elastic deformation was mainly carried out by the amorphous soft part of the PTFE matrix, which was mainly represented by the interlamellar slip in the amorphous region and this process was reversible [21]. However, the difference is that when the Ni volume fraction was less than 30%, the specimens (type A, B, C, D) went through an elastic deformation and a plastic deformation during compression and strain hardening occurred after yielding; yet when the Ni volume fraction was greater than 30%, the specimens (type E, F) only underwent a linear elastic stage and a failure stage and failed directly after reaching the yield strength. This is because the Ni filler played an important role in reinforcing the materials when the Ni content was low. However, excessive Ni would destroy the continuity of the PTFE matrix, leading to a reduction of the material’s strength, which can be confirmed in type F in the Figure 6.

The mechanical property parameters of the six types of specimens, calculated based on the stress-strain data, are illustrated in Table 3. As Table 3 lists, the addition of Ni particles to Al-PTFE had a significant effect on the mechanical properties of the material. As the Ni content rose, the strength of the material first increased and subsequently decreased. When the Ni volume fraction was 10%, the strain hardening modulus and compressive strength (which represents the strength of the material reached at the maximum) which were 51.35 MPa and 111.41 MPa respectively. Besides, the failure strain of the material decreased monotonously with the increase in Ni content, reaching a maximum value of 2.04 when the material was not added to Ni and a minimum value of 0.35 when the Ni volume fraction was 40%, indicating that the material changed from ductile to brittle with an increasing Ni content.

### 3.4. Reaction Characteristics under Quasi-Static Compression

**Figure 7:**
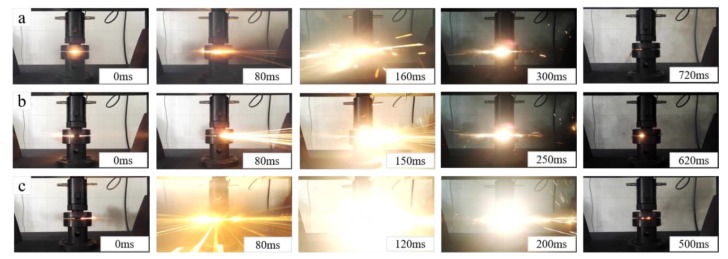
Reaction process of the type A, B, C specimens under quasi-static compression: (**a**) the type A specimen; (**b**) the type B specimen; (**c**) the type C specimen.

In the quasi-static compression test, it was observed that the type A, B, C specimens underwent a violent reaction and all of the specimens reacted completely, whereas no reaction occurred in the type D, E, F specimens. The reaction process of the type A, B, C specimens, captured using a high-speed camera, are shown in Figure 7. As Figure 7 indicates, the ignition phenomenon of the type C specimen turned out to be the most intense among the three. In contrast, the reaction intensity of the type A specimen was slightly weaker than that of the type B and C specimens. Apart from this, the reaction time of the type C specimen was the shortest (~500 ms), which was a little higher than that of the type A (~620 ms) and B (~720 ms) specimens. The conclusion that can be reached is that adding Ni to Al-PTFE can enhance the reaction intensity and reaction speed of the material under the pre-requisite that the Ni volume fraction is not more than 10%.

**Figure 8:**
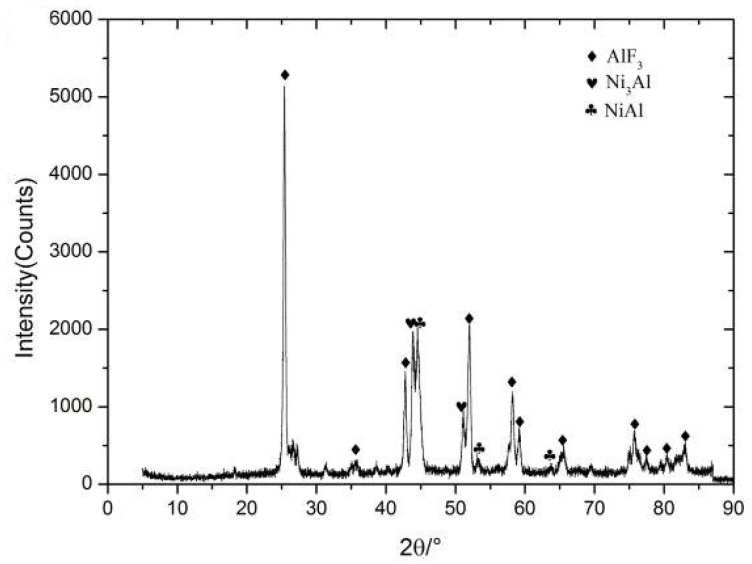
The X-ray diffraction result of the reaction residues of type C specimen after quasi-static compression.

To explain the experimental phenomenon in quasi-static compression and to understand the chemical reaction mechanism of Al-Ni-PTFE, the residues were characterized by X-ray diffraction. The X-ray diffraction results for the reaction residues of the type C specimen after quasi-static compression are presented in Figure 8. The results show that AlF_3_, Ni_3_Al, NiAl were produced during the quasi-static compression reaction of the type C specimen. On this basis, the chemical reaction process of Al-Ni-PTFE under quasi-static compression can be concluded as follows:

(1) $$\left\{ \begin{array}{l} 4\mathrm{Al}+3\left({-C}_{2}F_{4}-\right)\to 4{\mathrm{AlF}}_{3}+6C\;\\ \mathrm{Al}+3\mathrm{Ni}\to {\mathrm{Ni}}_{3}\mathrm{Al}\;\\ \mathrm{Al}+\mathrm{Ni}\to \mathrm{NiAl} \end{array}\right..$$

Arising from the absence of Al and Ni in the residues, it can be obtained that Al and Ni had reacted completely. Besides, there was no NiF_2_ in the resultant residue, indicating that there was no reaction between Ni and PTFE.

Feng put forward a crack-induced initiation mechanism after he found that Al-PTFE underwent a violent reaction under quasi-static compression. A simple description is that when the stress reaches the compressive strength of the Al-PTFE specimen, the crack tip generates local hot spots instantly stimulating the specimen to react [9]. By comparing the thermal behavior and mechanical properties of materials, analyzing the reaction phenomena in the quasi-static compression test, and linking the crack-induced initiation mechanism, the following conclusions can be drawn. On the one hand, for the type A, B, C specimens, which can react under quasi-static compression, by virtue of their higher strength and better toughness, the specimens could absorb more energy during the compression process and the hot spots at the crack tip could stimulate the reaction of Al and PTFE. The energy released by the reaction further excited the reaction of Al and Ni. According to the analysis of the thermal behavior, the reaction heat of Al-Ni was far higher than that of Al-PTFE under the same volume fraction. Therefore, the reaction of the type C specimen was more violent than that of the type A specimen. This further proves the feasibility of increasing the energy density and damage effect of Al-Ni-PTFE reactive material by means of the Ni-Al reaction. On the other hand, the type D, E, F specimens were not capable of reacting under quasi-static compression. Due to the high content of Ni, the material became brittle and the toughness was relatively low. The material failed directly after the compressive strength was reached and the energy absorbed was insufficient to generate local hot spots at the crack tip. Therefore, the Al and PTFE were not capable of reacting and a reaction between Al and Ni did not occur because of the lack of excitation of the reaction energy of Al and PTFE.

## 4. Conclusions

In this paper, the thermal behavior of Al-PTFE and an Al-Ni mixture was studied using the DSC method. The mechanical properties and reaction characteristics of six types of Al-Ni-PTFE specimens under quasi-static compression were ascertained. The conclusions can be drawn as follows:

(1) The TG-DSC curves of Al-PTFE and Al-Ni mixtures illustrated that the reaction between Al and PTFE began at 587.6 °C, the reaction between Al and Ni started at 582.7 °C, and the reaction heat of Al-Ni (991.9 J/g) was 12.5 times higher than that of Al-PTFE (79.6 J/g).
(2) The addition of Ni particles to Al-PTFE had a significant effect on the mechanical properties of the material. With the increase in Ni content, the material changed from ductile to brittle and the strain hardening modulus and compressive strength rose first and subsequently decreased, reaching a maximum of 51.35 MPa and 111.41 MPa respectively when the Ni volume fraction was 10%.
(3) In the quasi-static compression test, no reaction was occurred for specimens with a Ni volume fraction of 20, 30 and 40%, while an exothermic reaction occurred for specimens with a Ni volume fraction of 0, 5 and 10%. As the reaction heat of Al-Ni was far higher than that of Al-PTFE, the reaction intensity and reaction speed took on an upward trend as the Ni content increased.
(4) In the Al-Ni-PTFE system, the reaction between Al and PTFE preceded the reaction between Al and Ni and the feasibility of increasing the energy density and the damaging effect of the Al-Ni-PTFE reactive material by means of the Ni-Al reaction was proved.

## Figures and Tables

**Table 1:** The equivalence ratios and TMD (theoretical maximum density) of the six types of Al-Ni-PTFE specimens.

Type	Volume Fraction (vol %)			Mass Fraction (wt %)			TMD (g/cm^3^)
	Al	Ni	PTFE	Al	Ni	PTFE	
A	22%	0	78%	26.5%	0	73.5%	2.31
B	22%	5%	73%	22.8%	17.1%	60.2%	2.61
C	22%	10%	68%	20.2%	30.2%	49.6%	2.95
D	22%	20%	58%	16.4%	49.2%	34.4%	3.62
E	22%	30%	48%	13.8%	62.2%	24.0%	4.30
F	22%	40%	38%	11.9%	71.6%	16.4%	4.97

**Table 2:** The parameters for the endothermic and exothermic peaks in the DSC curves.

No.	Onset Temperature/(°C)	Peak Temperature/(°C)	End Temperature/(°C)	Heat Release/(J/g)
Endo-peak-A	328.9	339.5	349.6	−45.4
Endo-peak-B	502.2	566.8	585.7	−389.2
Exo-peak-C	587.6	590.2	642.1	79.6
Endo-peak-D	649.1	660.0	667.3	−73.5
Exo-peak-E	582.7	597.3	605.5	991.9

**Table 3:** Mechanical properties of the six types of Al-Ni-PTFE specimens under quasi-static compression.

Type	Yield Strength/MPa	Elastic Modulus/MPa	Hardening Modulus/MPa	Compressive Strength/MPa	Failure Strain
A	19.92	358.86	37.64	92.77	2.04
B	21.14	360.48	43.26	96.67	1.81
C	27.13	363.02	51.35	111.41	1.74
D	36.67	364.09	39.77	80.36	1.29
E	46.75	366.52	-	58.42	0.50
F	57.83	367.01	-	64.04	0.35
